# 2-Hy­droxy-*N*-(3-nitro­phen­yl)benzamide

**DOI:** 10.1107/S1600536810031910

**Published:** 2010-08-28

**Authors:** Abdul Rauf Raza, Bushra Nisar, M. Nawaz Tahir

**Affiliations:** a Department of Chemistry, University of Sargodha, Sargodha, Pakistan; b Department of Physics, University of Sargodha, Sargodha, Pakistan

## Abstract

In the crystal structure of title compound, C_13_H_10_N_2_O_4_, as expected, the nitro- and hy­droxy-substituted benzene rings are planar with r. m. s. deviations of 0.0037 and 0.0014 Å, respectively, but are twisted slightly relative to each other, making a dihedral angle of 12.23 (7)°. The nitro group is only slightly twisted [by 2.71 (16)°] with respect to its parent ring. An intra­molecular N—H⋯O hydrogen bond forms an *S*(6) ring motif. Inter­molecular N—H⋯O and O—H⋯O hydrogen bonds build up sheets parallel to the *ab* plane. Futhermore, weak π–π inter­actions [centroid–centroid distances = 3.7150 (8) 3.7342 (6) and 3.9421 (8) Å] between the rings yield a three-dimensional network.

## Related literature

For the pharmaceutical properties of benzoxazepines and their derivatives, see: Fattorusso *et al.* (2005 ▶); Samanta *et al.* (2010 ▶). For related structures, see: Raza *et al.* (2009 ▶, 2010 ▶); Glidewell *et al.* (2006 ▶). For hydrogen-bonding discussion, see: Bernstein *et al.* (1995 ▶); Janiak (2000 ▶).
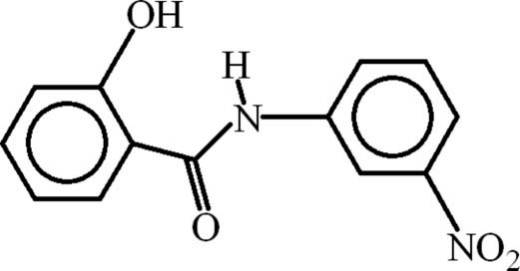


## Experimental

### 

#### Crystal data

C_13_H_10_N_2_O_4_
*M* *_r_* = 258.23
Monoclinic, 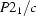

*a* = 7.8385 (2) Å
*b* = 11.9531 (3) Å
*c* = 12.3550 (3) Å
β = 90.860 (1)°
*V* = 1157.46 (5) Å^3^
*Z* = 4
Mo *K*α radiation
μ = 0.11 mm^−1^
*T* = 296 K
0.28 × 0.22 × 0.20 mm

#### Data collection

Bruker Kappa APEXII CCD diffractometer
Absorption correction: multi-scan (*SADABS*; Bruker, 2009 ▶) *T*_min_ = 0.979, *T*_max_ = 0.988
4381 measured reflections
2874 independent reflections
2254 reflections with *I* > 2σ(*I*)
*R*_int_ = 0.023

#### Refinement

*R*[*F* ^2^ > 2σ(*F* ^2^)] = 0.041
*wR*(*F* ^2^) = 0.115
*S* = 1.03
2874 reflections
173 parameters
H-atom parameters constrained
Δρ_max_ = 0.26 e Å^−3^
Δρ_min_ = −0.19 e Å^−3^

### 

Data collection: *APEX2* (Bruker, 2009 ▶); cell refinement: *SAINT* (Bruker, 2009 ▶); data reduction: *SAINT*; program(s) used to solve structure: *SHELXS97* (Sheldrick, 2008 ▶); program(s) used to refine structure: *SHELXL97* (Sheldrick, 2008 ▶); molecular graphics: *ORTEP-3 for Windows* (Farrugia, 1997 ▶) and *PLATON* (Spek, 2009 ▶); software used to prepare material for publication: *WinGX* (Farrugia, 1999 ▶) and *PLATON*.

## Supplementary Material

Crystal structure: contains datablocks global, I. DOI: 10.1107/S1600536810031910/dn2595sup1.cif

Structure factors: contains datablocks I. DOI: 10.1107/S1600536810031910/dn2595Isup2.hkl

Additional supplementary materials:  crystallographic information; 3D view; checkCIF report

## Figures and Tables

**Table 1:** Hydrogen-bond geometry (Å, °)

*D*—H⋯*A*	*D*—H	H⋯*A*	*D*⋯*A*	*D*—H⋯*A*
N1—H1⋯O4	0.86	1.98	2.6450 (14)	133
N1—H1⋯O1^i^	0.86	2.50	3.1381 (16)	132
O4—H4*A*⋯O3^ii^	0.82	1.83	2.6488 (13)	174

Symmetry codes: (i) 
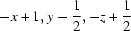
; (ii) 
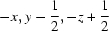
.

**Fig. 1.**
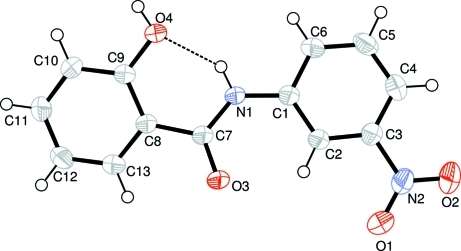
View of the title compound with the atom numbering scheme. The thermal displacements are drawn at the 50% probability level. H-atoms are shown by small circles of arbitrary radii. The dotted line indicate the intramolecular H-bond.

**Fig. 2.**
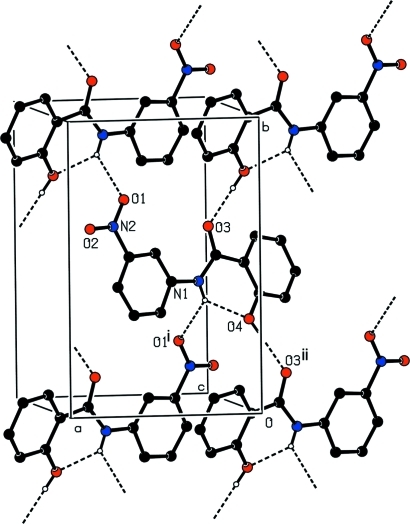
Partial packing view showing the two dimensional polymeric network parallel to ab-plane. H atoms not involved in hydrogen bondings have been omitted for clarity. H bonds are shown as dashed lines. [Symmetry codes: (i) -x+1, y-1/2, -z+1/2; (ii) -x, y-1/2, -z+1/2].

## supplementary crystallographic information

### Comment

Asymmetric synthesis is gaining much importance. Aim of our work is the formation of various chiral benzoxazepines and their derivatives that have been reported as anti-tumor (Samanta *et al.*, 2010) and anti-HIV agents (Fattorusso *et al.*, 2005). The title compound (I, Fig.1) has been synthesized as a precursor for variously substituted chiral benzoxazepines.

We have reported the crystal structures of (II) *i.e.*, 2-hydroxy-3-nitro-*N*-phenylbenzamide (Raza *et al.*, 2009) and (III) 2-hydroxy-5-nitro-*N*-phenylbenzamide (Raza *et al.*, 2010). The title compound differs from (II) and (III) due to the attachement of nitro group at different position.

In (I), the nitro and hydroxy substituted phenyl rings A (C1–C6) and B (C8—C13) are planar with r. m. s. deviation of 0.0037 and 0.0014 Å, respectively. The central group C (N1/C7/O3) is of course planar. The dihedral angle between A/B, A/C and B/C is 12.23 (7)°, 6.13 (20)° and 18.35 (18)°, respectively. The nitro group is slightly twisted with respect to its parent phenyl ring making a dihedral angle of 2.71 (16)°. Bond distances and angles agree with related compounds (Raza *et al.*, 2009,2010; Glidewell *et al.*, 2006).

There exist intramolecular N—H···O hydrogen bond forming S(6) ring motifs (Bernstein *et al.*, 1995). The molecules are stabilized in the form of two dimensional polymeric sheets due to intermolecular H-bondings of N—H···O and O—H···O types. The polymeric sheets extend in the *ab*-plane (Table 1, Fig. 2). Futhermore weak slippest π–π interactions between the phenyl rings yield a three dimensionnal network (Table 2).

### Experimental

3-Nitroaniline (4.14 g, 0.03 mol) was added to 2-hydroxybenzoyl chloride prepared by treating salicylic acid (4.14 g, 0.03 mol) with oxalyl chloride (2.80 ml, 4.00 g, 0.032 mol) using DMF in catalytic amount. The reaction mixture was refluxed for 2 h, cooled to room temperature, neutralized with aqueous NaHCO3 (10%) and extracted with EtOAc (3×25 ml). The organic extract was combined, dried over anhydrous Na_2_SO_4_, filtered and concentrated under reduced pressure. The title compound (I) was mechanically separated as yellow cubical crystals.

### Refinement

Although H atoms were appeared in difference Fourier map but were positioned geometrically with (O–H = 0.82, N–H= 0.86 and C–H = 0.93 Å) and refined as riding with *U*_iso_(H) = *xU*_eq_(C, N, O), where *x* = 1.5 for hydroxy and *x* = 1.2 for other H atoms.

### Crystal data

**Table:**

C_13_H_10_N_2_O_4_	*F*(000) = 536
*M**_r_* = 258.23	*D*_x_ = 1.482 Mg m^−^^3^
Monoclinic, *P*2_1_/*c*	Mo *K*α radiation, λ = 0.71073 Å
Hall symbol: -P 2ybc	Cell parameters from 931 reflections
*a* = 7.8385 (2) Å	θ = 2.8–26.0°
*b* = 11.9531 (3) Å	µ = 0.11 mm^−^^1^
*c* = 12.3550 (3) Å	*T* = 296 K
β = 90.860 (1)°	Prisms, orange
*V* = 1157.46 (5) Å^3^	0.28 × 0.22 × 0.20 mm
*Z* = 4	

### Data collection

**Table:**

Bruker Kappa APEXII CCD diffractometer	2874 independent reflections
Radiation source: fine-focus sealed tube	2254 reflections with *I* > 2σ(*I*)
graphite	*R*_int_ = 0.023
Detector resolution: 7.5 pixels mm^-1^	θ_max_ = 28.3°, θ_min_ = 2.4°
ω scans	*h* = −10→8
Absorption correction: multi-scan (*SADABS*; Bruker, 2009)	*k* = −15→15
*T*_min_ = 0.979, *T*_max_ = 0.988	*l* = −16→15
4381 measured reflections	

### Refinement

**Table:**

Refinement on *F*^2^	Primary atom site location: structure-invariant direct methods
Least-squares matrix: full	Secondary atom site location: difference Fourier map
*R*[*F*^2^ > 2σ(*F*^2^)] = 0.041	Hydrogen site location: inferred from neighbouring sites
*wR*(*F*^2^) = 0.115	H-atom parameters constrained
*S* = 1.03	*w* = 1/[σ^2^(*F*_o_^2^) + (0.0525*P*)^2^ + 0.2825*P*] where *P* = (*F*_o_^2^ + 2*F*_c_^2^)/3
2874 reflections	(Δ/σ)_max_ < 0.001
173 parameters	Δρ_max_ = 0.26 e Å^−^^3^
0 restraints	Δρ_min_ = −0.19 e Å^−^^3^

### Special details

**Table:**

Geometry. Bond distances, angles *etc*. have been calculated using the rounded fractional coordinates. All su's are estimated from the variances of the (full) variance-covariance matrix. The cell e.s.d.'s are taken into account in the estimation of distances, angles and torsion angles
Refinement. Refinement of *F*^2^ against ALL reflections. The weighted *R*-factor *wR* and goodness of fit *S* are based on *F*^2^, conventional *R*-factors *R* are based on *F*, with *F* set to zero for negative *F*^2^. The threshold expression of *F*^2^ > σ(*F*^2^) is used only for calculating *R*-factors(gt) *etc*. and is not relevant to the choice of reflections for refinement. *R*-factors based on *F*^2^ are statistically about twice as large as those based on *F*, and *R*- factors based on ALL data will be even larger.

### Fractional atomic coordinates and isotropic or equivalent isotropic displacement parameters (Å^2^)

**Table:**

	*x*	*y*	*z*	*U*_iso_*/*U*_eq_	
O1	0.67761 (15)	0.72384 (9)	0.11659 (11)	0.0599 (4)	
O2	0.87762 (16)	0.63301 (11)	0.04018 (13)	0.0864 (6)	
O3	0.19790 (13)	0.61776 (7)	0.25603 (10)	0.0529 (3)	
O4	−0.01040 (12)	0.30182 (7)	0.25612 (9)	0.0435 (3)	
N1	0.25743 (14)	0.43458 (8)	0.23703 (10)	0.0406 (4)	
N2	0.74358 (15)	0.63616 (11)	0.08799 (10)	0.0478 (4)	
C1	0.41415 (16)	0.43895 (10)	0.18388 (11)	0.0340 (4)	
C2	0.50276 (16)	0.53731 (10)	0.16487 (11)	0.0354 (4)	
C3	0.65510 (16)	0.53059 (11)	0.11058 (11)	0.0361 (4)	
C4	0.72509 (18)	0.43172 (12)	0.07522 (12)	0.0430 (4)	
C5	0.63600 (18)	0.33434 (12)	0.09615 (12)	0.0445 (4)	
C6	0.48277 (18)	0.33755 (11)	0.14922 (11)	0.0398 (4)	
C7	0.15642 (15)	0.51916 (9)	0.26848 (11)	0.0338 (4)	
C8	−0.00575 (15)	0.48872 (10)	0.32268 (10)	0.0325 (3)	
C9	−0.08272 (16)	0.38269 (10)	0.31694 (10)	0.0334 (3)	
C10	−0.23250 (17)	0.36262 (11)	0.37214 (12)	0.0420 (4)	
C11	−0.30562 (19)	0.44557 (12)	0.43280 (13)	0.0485 (5)	
C12	−0.23231 (19)	0.55075 (12)	0.43935 (12)	0.0455 (4)	
C13	−0.08404 (17)	0.57126 (11)	0.38488 (11)	0.0388 (4)	
H1	0.22076	0.36854	0.25156	0.0487*	
H2	0.46071	0.60586	0.18806	0.0424*	
H4	0.82802	0.43043	0.03876	0.0516*	
H4A	−0.06998	0.24546	0.25731	0.0652*	
H5	0.68008	0.26593	0.07413	0.0534*	
H6	0.42427	0.27130	0.16212	0.0477*	
H10	−0.28373	0.29254	0.36804	0.0504*	
H11	−0.40553	0.43091	0.46989	0.0582*	
H12	−0.28277	0.60681	0.48010	0.0546*	
H13	−0.03449	0.64182	0.38951	0.0465*	

### Atomic displacement parameters (Å^2^)

**Table:**

	*U*^11^	*U*^22^	*U*^33^	*U*^12^	*U*^13^	*U*^23^
O1	0.0605 (7)	0.0384 (6)	0.0814 (8)	−0.0136 (5)	0.0186 (6)	−0.0046 (5)
O2	0.0610 (8)	0.0748 (9)	0.1249 (13)	−0.0213 (7)	0.0524 (8)	−0.0141 (8)
O3	0.0429 (5)	0.0216 (4)	0.0950 (8)	0.0011 (4)	0.0234 (5)	0.0044 (5)
O4	0.0406 (5)	0.0258 (4)	0.0646 (6)	−0.0064 (4)	0.0155 (4)	−0.0098 (4)
N1	0.0381 (6)	0.0205 (5)	0.0636 (8)	−0.0002 (4)	0.0166 (5)	0.0025 (5)
N2	0.0407 (6)	0.0484 (7)	0.0546 (7)	−0.0111 (5)	0.0106 (5)	−0.0035 (6)
C1	0.0324 (6)	0.0275 (6)	0.0421 (7)	0.0017 (4)	0.0040 (5)	0.0012 (5)
C2	0.0326 (6)	0.0278 (6)	0.0459 (7)	0.0016 (5)	0.0043 (5)	−0.0017 (5)
C3	0.0321 (6)	0.0364 (7)	0.0399 (7)	−0.0026 (5)	0.0014 (5)	−0.0004 (5)
C4	0.0359 (7)	0.0490 (8)	0.0443 (7)	0.0064 (6)	0.0078 (5)	−0.0028 (6)
C5	0.0491 (8)	0.0358 (7)	0.0489 (8)	0.0123 (6)	0.0063 (6)	−0.0053 (6)
C6	0.0451 (7)	0.0273 (6)	0.0471 (7)	0.0035 (5)	0.0053 (6)	−0.0010 (5)
C7	0.0321 (6)	0.0239 (6)	0.0456 (7)	0.0005 (4)	0.0046 (5)	0.0016 (5)
C8	0.0329 (6)	0.0250 (6)	0.0398 (6)	0.0002 (4)	0.0035 (5)	0.0019 (5)
C9	0.0340 (6)	0.0259 (6)	0.0405 (6)	0.0007 (5)	0.0038 (5)	−0.0007 (5)
C10	0.0382 (7)	0.0316 (6)	0.0564 (8)	−0.0060 (5)	0.0102 (6)	0.0003 (6)
C11	0.0417 (7)	0.0442 (8)	0.0603 (9)	0.0001 (6)	0.0209 (7)	0.0005 (7)
C12	0.0483 (8)	0.0367 (7)	0.0520 (8)	0.0073 (6)	0.0154 (6)	−0.0040 (6)
C13	0.0421 (7)	0.0272 (6)	0.0472 (7)	0.0012 (5)	0.0058 (6)	−0.0015 (5)

### Geometric parameters (Å, °)

**Table:**

O1—N2	1.2232 (17)	C7—C8	1.4907 (17)
O2—N2	1.2136 (18)	C8—C13	1.3981 (18)
O3—C7	1.2329 (14)	C8—C9	1.4050 (17)
O4—C9	1.3540 (15)	C9—C10	1.3875 (19)
O4—H4A	0.8200	C10—C11	1.373 (2)
N1—C1	1.4026 (17)	C11—C12	1.384 (2)
N1—C7	1.3451 (15)	C12—C13	1.374 (2)
N2—C3	1.4687 (18)	C2—H2	0.9300
N1—H1	0.8600	C4—H4	0.9300
C1—C6	1.3959 (18)	C5—H5	0.9300
C1—C2	1.3874 (17)	C6—H6	0.9300
C2—C3	1.3807 (18)	C10—H10	0.9300
C3—C4	1.3768 (19)	C11—H11	0.9300
C4—C5	1.384 (2)	C12—H12	0.9300
C5—C6	1.378 (2)	C13—H13	0.9300
			
O1···O4^i^	3.1660 (16)	C10···C5^v^	3.564 (2)
O1···N1^i^	3.1381 (16)	C10···O3^iv^	3.3407 (17)
O3···C10^ii^	3.3407 (17)	C12···C13^x^	3.582 (2)
O3···C9^ii^	3.4105 (15)	C12···C8^x^	3.4906 (19)
O3···O4^ii^	2.6488 (13)	C13···C13^x^	3.5521 (19)
O3···C5^i^	3.4148 (18)	C13···O4^ii^	3.3491 (16)
O3···C2	2.8257 (16)	C13···C12^x^	3.582 (2)
O4···N1	2.6450 (14)	C7···H4A^ii^	2.8100
O4···O1^iii^	3.1660 (16)	C7···H2	2.8000
O4···C13^iv^	3.3491 (16)	C9···H1	2.5300
O4···O3^iv^	2.6488 (13)	C10···H5^xi^	3.0200
O4···C4^v^	3.4009 (18)	C11···H11^xii^	2.9700
O4···C5^v^	3.4022 (18)	C11···H5^xi^	3.0800
O1···H12^vi^	2.6600	C12···H11^xii^	3.0800
O1···H2	2.3900	C13···H4A^ii^	2.9900
O1···H1^i^	2.5000	H1···O4	1.9800
O1···H6^i^	2.9200	H1···C9	2.5300
O2···H4	2.4500	H1···H6	2.2700
O2···H4^vii^	2.6300	H1···O1^iii^	2.5000
O3···H13	2.4900	H2···O1	2.3900
O3···H10^ii^	2.6800	H2···O3	2.2400
O3···H2	2.2400	H2···C7	2.8000
O3···H4A^ii^	1.8300	H4···O2	2.4500
O3···H5^i^	2.9000	H4···O2^vii^	2.6300
O4···H1	1.9800	H4A···H10	2.2500
O4···H13^iv^	2.6500	H4A···O3^iv^	1.8300
N1···O4	2.6450 (14)	H4A···C7^iv^	2.8100
N1···O1^iii^	3.1381 (16)	H4A···C13^iv^	2.9900
N2···C6^viii^	3.4176 (18)	H4A···H13^iv^	2.3500
C2···O3	2.8257 (16)	H5···O3^iii^	2.9000
C2···C4^viii^	3.459 (2)	H5···C10^xiii^	3.0200
C4···O4^ix^	3.4009 (18)	H5···C11^xiii^	3.0800
C4···C9^ix^	3.3759 (19)	H6···H1	2.2700
C4···C2^viii^	3.459 (2)	H6···O1^iii^	2.9200
C5···O4^ix^	3.4022 (18)	H10···H4A	2.2500
C5···C9^ix^	3.5293 (19)	H10···O3^iv^	2.6800
C5···C10^ix^	3.564 (2)	H11···C11^xii^	2.9700
C5···O3^iii^	3.4148 (18)	H11···C12^xii^	3.0800
C6···C10^ix^	3.532 (2)	H11···H11^xii^	2.3500
C6···N2^viii^	3.4176 (18)	H11···H12^xii^	2.5700
C8···C12^x^	3.4906 (19)	H12···H11^xii^	2.5700
C9···C4^v^	3.3759 (19)	H12···O1^xiv^	2.6600
C9···C5^v^	3.5293 (19)	H13···O3	2.4900
C9···O3^iv^	3.4105 (15)	H13···O4^ii^	2.6500
C10···C6^v^	3.532 (2)	H13···H4A^ii^	2.3500
			
C9—O4—H4A	109.00	C8—C9—C10	119.81 (11)
C1—N1—C7	129.11 (10)	O4—C9—C8	119.27 (11)
O1—N2—O2	122.69 (13)	O4—C9—C10	120.92 (11)
O2—N2—C3	118.72 (13)	C9—C10—C11	120.43 (12)
O1—N2—C3	118.58 (12)	C10—C11—C12	120.75 (14)
C7—N1—H1	115.00	C11—C12—C13	119.14 (13)
C1—N1—H1	115.00	C8—C13—C12	121.66 (12)
N1—C1—C2	123.71 (11)	C1—C2—H2	121.00
N1—C1—C6	117.10 (11)	C3—C2—H2	121.00
C2—C1—C6	119.19 (12)	C3—C4—H4	121.00
C1—C2—C3	118.15 (11)	C5—C4—H4	121.00
N2—C3—C2	117.17 (11)	C4—C5—H5	120.00
N2—C3—C4	119.05 (12)	C6—C5—H5	120.00
C2—C3—C4	123.76 (12)	C1—C6—H6	120.00
C3—C4—C5	117.27 (13)	C5—C6—H6	120.00
C4—C5—C6	120.77 (13)	C9—C10—H10	120.00
C1—C6—C5	120.86 (12)	C11—C10—H10	120.00
N1—C7—C8	117.13 (10)	C10—C11—H11	120.00
O3—C7—C8	121.17 (11)	C12—C11—H11	120.00
O3—C7—N1	121.67 (11)	C11—C12—H12	120.00
C7—C8—C9	124.47 (11)	C13—C12—H12	120.00
C7—C8—C13	117.32 (11)	C8—C13—H13	119.00
C9—C8—C13	118.20 (11)	C12—C13—H13	119.00
			
C7—N1—C1—C2	7.6 (2)	C4—C5—C6—C1	−0.5 (2)
C7—N1—C1—C6	−172.64 (13)	O3—C7—C8—C9	163.67 (13)
C1—N1—C7—O3	−2.8 (2)	O3—C7—C8—C13	−17.53 (19)
C1—N1—C7—C8	179.24 (12)	N1—C7—C8—C9	−18.36 (19)
O1—N2—C3—C2	−1.05 (19)	N1—C7—C8—C13	160.43 (12)
O1—N2—C3—C4	177.63 (13)	C7—C8—C9—O4	−2.03 (19)
O2—N2—C3—C2	−179.85 (14)	C7—C8—C9—C10	178.87 (12)
O2—N2—C3—C4	−1.2 (2)	C13—C8—C9—O4	179.19 (12)
N1—C1—C2—C3	−179.31 (13)	C13—C8—C9—C10	0.09 (18)
C6—C1—C2—C3	1.0 (2)	C7—C8—C13—C12	−178.94 (13)
N1—C1—C6—C5	179.86 (13)	C9—C8—C13—C12	−0.1 (2)
C2—C1—C6—C5	−0.4 (2)	O4—C9—C10—C11	−179.40 (13)
C1—C2—C3—N2	177.89 (12)	C8—C9—C10—C11	−0.3 (2)
C1—C2—C3—C4	−0.7 (2)	C9—C10—C11—C12	0.5 (2)
N2—C3—C4—C5	−178.69 (13)	C10—C11—C12—C13	−0.5 (2)
C2—C3—C4—C5	−0.1 (2)	C11—C12—C13—C8	0.3 (2)
C3—C4—C5—C6	0.7 (2)		

Symmetry codes: (i) −*x*+1, *y*+1/2, −*z*+1/2; (ii) −*x*, *y*+1/2, −*z*+1/2; (iii) −*x*+1, *y*−1/2, −*z*+1/2; (iv) −*x*, *y*−1/2, −*z*+1/2; (v) *x*−1, *y*, *z*; (vi) *x*+1, −*y*+3/2, *z*−1/2; (vii) −*x*+2, −*y*+1, −*z*; (viii) −*x*+1, −*y*+1, −*z*; (ix) *x*+1, *y*, *z*; (x) −*x*, −*y*+1, −*z*+1; (xi) *x*−1, −*y*+1/2, *z*+1/2; (xii) −*x*−1, −*y*+1, −*z*+1; (xiii) *x*+1, −*y*+1/2, *z*−1/2; (xiv) *x*−1, −*y*+3/2, *z*+1/2.

### Hydrogen-bond geometry (Å, °)

**Table:**

*D*—H···*A*	*D*—H	H···*A*	*D*···*A*	*D*—H···*A*
N1—H1···O4	0.86	1.98	2.6450 (14)	133
N1—H1···O1^iii^	0.86	2.50	3.1381 (16)	132
O4—H4A···O3^iv^	0.82	1.83	2.6488 (13)	174

Symmetry codes: (iii) −*x*+1, *y*−1/2, −*z*+1/2; (iv) −*x*, *y*−1/2, −*z*+1/2.

### Table 2 π-π contacts (Å, °)

**Table:**

ring 1/ring 2	ccd(Å)	ipd(Å)	sa(°)
Cg1->Cg2^i^	3.7150 (8)	3.455	21.6
Cg3->Cg4^ii^	3.7342 (6)	3.539	18.6
Cg5->Cg5^iii^	3.9421 (8)	3.443	29.1

Symmetry codes: (i) 1-x,1-y,-z, (ii) 1+x,y,z, (iii) -x,1-y,1-zCg1:  C1,C2,C3,C4,C5,C6Cg2:  C8,C9,C10,C11,C12,C13ccd: center-to-center distance (Distance between ring centroids);ipd: mean interplanar distance (Distance from one plane to the neighbouring centroid);sa: mean slippage angle (Angle subtended by the intercentroid vector to the plane normal).For details, see Janiak (2000)
